# High-Temperature Fatigue Degradation Behaviors of a 3D Braided C/SiC with a Thin Interlayer in Different Dry Oxygen Atmospheres

**DOI:** 10.3390/ma17194925

**Published:** 2024-10-09

**Authors:** Lexin Yang, Dianwei He, Chen Hu, Zhenhuan Gao, Liping Nie, Youbei Sun, Lei Zhang, Xingang Luan

**Affiliations:** 1 State Key Laboratory of Clean and Efficient Turbomachinery Power Equipment, Deyang 618000, China; ylx599@163.com (L.Y.); gaozhenhuan@dongfang.com (Z.G.); nieliping@dongfang.com (L.N.); richonf@163.com (Y.S.); 2 Science and Technology on Thermostructural Composite Materials Laboratory, School of Materials Science and Engineering, Northwestern Polytechnical University, Xi’an 710072, China; 13983788698@163.com (D.H.); 18363380455@163.com (C.H.); 3 Dongfang Electric Corporation Dongfang Turbine Co., Ltd., Deyang 618000, China; 4 School of Information Engineering, Hangzhou Dianzi University, Hangzhou 310018, China; zhanglei@hdu.edu.cn

**Keywords:** C/SiC, thin interlayer, fatigue, oxygen partial pressure, oxidation

## Abstract

In order to evaluate the increase in the flexural strength of a 3D braided C/SiC composite comprised with a thin pyrolytic carbon (PyC) interlayer (TI C/SiC) under a load of 60 MPa with an amplitude of ±20 MPa at an oxygen partial pressure of 8000 Pa, the effect of temperature, oxidation and stress value on the length change in the sample, fracture behavior, residual flexural strength and fracture morphology were studied up to 1500 °C. It was found that the gauge length change behaviors of the material are related to (i) the positive damage of the thin interlayer and (ii) to the negative damage of the C phase. The most serious damage of TI C/SiC under 60 ± 20 MPa occurs in an oxygen partial pressure of 17,000 Pa at 1300 °C. When the oxygen partial pressure and/or the temperature are reduced, the positive C phase damage is relieved. In the case that the oxygen partial pressure, temperature and stress increase, the negative C phase damage is facilitated. The oxidation mechanism of the C phase is controlled by the inward diffusion of oxygen from the sample surface to the center; however, a higher stress is considered to change the oxygen diffusion mechanism by increasing the reaction of the C phase, with oxygen causing a widening of microcracks.

## 1. Introduction

Ceramic matrix composites (CMCs) have been anticipated to serve as high-temperature structural components for aeroengines, hypersonic and rocket engines due to their low density and high specific strength over a large temperature range. Moreover, CMCs show excellent damage tolerance and high potential in reducing component weights and cooling flow compared to current nickel-base superalloys and monolithic ceramics [1]. In this regard, 3D braided SiC/SiC provide the best 500 h lasting rupture strength. However, SiC fiber cannot be used for longtime applications beyond 1400 °C [2]. C/SiC exhibits higher temperature resistance, rendering it more suitable for hypersonic and rocket engine applications, because the C fiber is the only one that can tolerate temperature above 1500 °C. Among C/SiC materials, the 3D braided one also exhibits the highest fatigue limits at room temperature and up to 1500 °C under 10^−4^ Pa vacuum [3]. Consequently, 3D braided C/SiC has been identified as the most promising candidate composite for the aforementioned applications [4,5,6,7,8].

For hypersonic applications, devices are exposed to environments characterized by high-temperature gas flow with low oxygen partial pressures and various loads, namely creep and fatigue stress [9]. Currently, the fatigue damage mechanism and life prediction method of 3D braided C/SiC at room temperature or high temperatures in vacuum have been intensively investigated based on the evolution of microstructure and deformation properties [10,11,12]. While one study has been performed in an oxygen partial pressure of 8000 Pa [13], less information on the fatigue degradation behaviors of 3D braided C/SiC in high-temperature gas with low oxygen partial pressures is available in the literature.

Furthermore, most of the reported 3D braided C/SiC composites have an interlayer thickness of 200–500 nm, which is optimized in terms of room temperature mechanical properties. However, it remains unclear whether this interlayer thickness is also an ideal choice with respect to improving the fatigue oxidation behavior of 3D braided C/SiC. Our previous work [14] about the fatigue oxidation behavior of the 3D braided C/SiC composite in aero-engine atmospheres indicated that the 3D braided C/SiC with a thin interlayer (TI C/SiC) showed enhanced anti-fatigue properties in an oxidative atmosphere. This behavior is due to the fact that appropriate interface damage improved the flexural strength of the composite.

It should be noted from our previous work [14] that the strength improvement in TI C/SiC mainly occurred after tested in an oxygen partial pressure of 8000 Pa. As it is a promising advanced material for hypersonic applications, whether the interface damage is the only reason for the strength improvement or whether other factors have to be taken into account has to be examined.

Consequently, the fatigue degradation behavior of TI C/SiC in dry oxygen was investigated in more detail in this study. In this context, the influence of temperature, oxygen partial pressure and stress value on the fatigue degradation is discussed in order to confirm the dominant environmental factors.

## 2. Experimental Section

### 2.1. Materials

**Figure 1:**
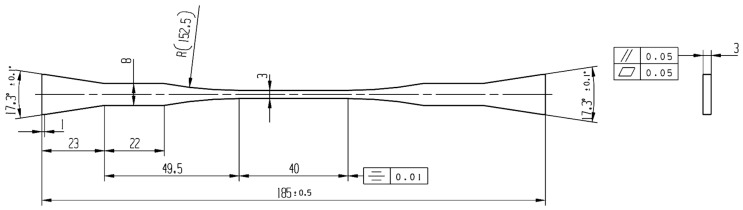
Shape and dimension of dog bone-shaped specimens (all sizes in mm).

**Figure 2:**
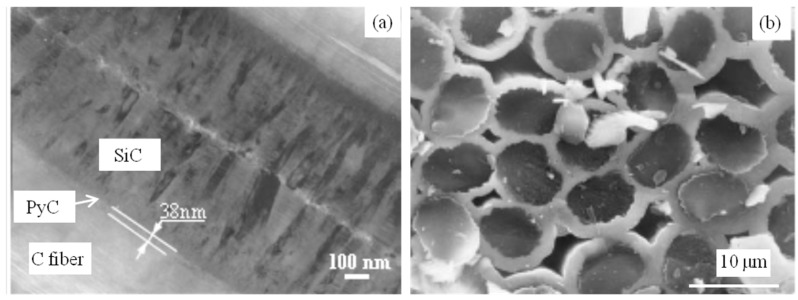
SEM images of the microstructure of the as-received TI C/SiC composite: (**a**) C fiber/SiC interlayer; (**b**) tensile fracture surface [14].

The 3D braided C/SiC was fabricated using the following procedures. First, fiber preforms were created of carbon fibers (T-300 Toray, Tokyo, Japan) using the three-dimensional braid method. The fiber volume fraction in the preform was ~40%. Then, low-pressure chemical vapor infiltration (LPCVI) was used to deposit pyrolytic carbon (PyC) at 960 °C as an interlayer using butane as the precursor, and then to deposit SiC at 1000 °C as a matrix using methyltrichlorosilane (MTS) and H_2_ (the flow rate of 150 mL/min) as source materials with a H_2_/MTS molar ratio of 10. The thickness of the PyC interlayer was controlled through the deposition time; the SiC matrix was prepared for 6~8 cycles until the density and the porosity reached about 2.0 g/cm^3^ and 10~15 vol%, respectively. Dog bone-shaped specimens (as shown in Figure 1) were cut from the prepared composite plates. The sample was redesigned based on the standard of ASTM C1359-13 [15] to avoid an unexpected break outside the gauge during fatigue. The 3 mm width of the gauge part was designed to minimize the effect of the other parts on the length change in the sample. A two-layer SiC coating was then deposited to seal the open ends of the fibers and open pores. The thickness of each SiC coating was 40 ± 5 µm. Finally, TI C/SiC samples with a thin PyC interlayer of 38 nm, C fiber volume fraction of ~40% and SiC matrix volume fraction of 45~50% were obtained. As reported in reference [14], the samples had a typical brittle fracture morphology, room temperature tensile strength of 154 ± 10 MPa (i.e., mean strength ± strength error) and room temperature bending strength of 283 ± 32 MPa (i.e., mean strength ± strength error). The microstructure of the as-received TI C/SiC sample is shown in Figure 2 [14], including the interlayer morphology (Figure 2a) and tensile fracture section morphology (Figure 2b).

### 2.2. Testing

Fatigue tests were conducted in an equivalent experimental simulating subsystem [14]. The system includes a Servo-Hydraulic testing device (Instron 8801, Instron Ltd., High Wycombe, UK) for loading samples, a high-temperature environment chamber for atmosphere control and an acquisition system for damage information such as electric resistance and sample deformation. During the heating process, the sample was fixed in the furnace and heated at the same time. After the furnace and the sample were heated to the settled temperature for 15 min, the test started, and the length change was recorded from zero. The length change in the gauge was evaluated using the displacement of the Instron’s lower loading cell according to reference [14]. The positive value of the displacement indicates a length increase, and the negative value means that the length was shortened. Only the largest length of each fatigue cycle was chosen to draw the length change curve of the sample, displaying the fatigue behavior.

The fatigue stresses were set at 60 ± 20 MPa (i.e., mean stress ± stress amplitude) and 90 ± 30 MPa (i.e., mean stress ± stress amplitude) with a frequency of 1 Hz; the related normalized mean stresses (defined as the ratio of applied mean stress to ultimate tensile strength) were 0.39 and 0.58, respectively. The fatigue tests were carried out at temperatures of 1000 °C, 1300 °C and 1500 °C in different dry oxygen/argon atmospheres, with different oxygen partial pressures of 0 Pa, 8000 Pa, 14,000 Pa and 21,000 Pa. During the tests, the total pressure of the environmental chamber was maintained at 100 kPa, and the gas flow was stable with a low velocity. After the sample was broken during the test, the gas was changed to Ar immediately to protect the fracture cross-section from oxidation. The residual three-point bending strength of the remaining gauge was tested using a universal testing machine (Instron 1196) with a loading rate of 0.5 mm/min. If the sample did not break after testing for a certain time, the residual three-point bending strength of the gauge was determined also using a universal testing machine (Instron 1196) with a loading rate of 0.5 mm/min. Young’s modulus was determined according to the slope of the first linear part of the displacement–load curve. The microstructure was observed using a field emission scanning electron microscope (FESEM, S4700, Hitachi, Tokyo, Japan).

## 3. Results and Discussions

### 3.1. Effect of Temperature on the Fatigue Behavior

**Figure 3:**
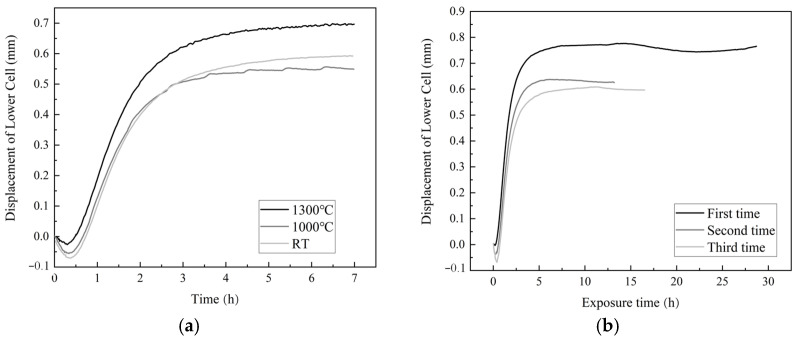

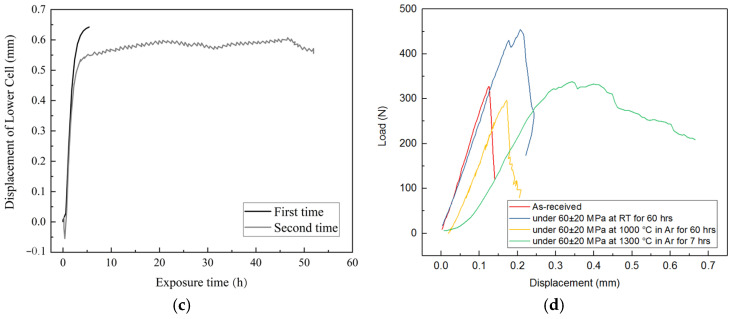
The behaviors of TI C/SiC under 60 ± 20 MPa in Ar at different temperatures (**a**), the length change during within 7 h at different temperatures, (**b**) the length change during 60 h at RT, (**c**) the length change during 60 h at 1000 °C, and (**d**) the flexural load‒displacement curves of the samples after fatigue at different temperatures and times.

The fatigue behavior of the composite TI C/SiC under 60 ± 20 MPa in Ar at different temperatures is shown in Figure 3. The length changes in the samples analyzed under the above-mentioned load at 1000 °C and 1300 °C for 7 h were compared to that at room temperature (RT), as shown in Figure 3a. The sample exhibits a smooth elongation curve in the second stage at RT during the first 7 h. The total length change at 1000 °C is comparable to that at RT, with more frequent length adjustment. However, the change in length at 1300 °C is higher, and the length adjustment is faster but less. In order to determine the inheritance and periodicity of the length change, the total 60 h test at RT was performed in three steps with three different holding times at the load; the curves are denoted as the first step, second step and third step, as illustrated in Figure 3b. According to the three curves in Figure 3b, it was found that the length exhibited a gradual reduction and subsequent elongation during the fatigue tested for 60 h. Additionally, the inheritance and periodicity of the length change was observed during the multiple tests. The initial shortening was found to be less and less with increasing testing time, indicating that the load-bearing area remained more stable after fatigue. The total 60 h test at 1000 °C was completed in two steps, as displayed in Figure 3c. In contrast to the prolonged adjustment at RT, the length change at 1000 °C demonstrates not only prolonged adjustment but also rapid short-term adjustment during the 60 h test (see Figure 3c). As illustrated in Figure 3d, the distinct length change behavior results in a disparate flexural deformation behavior of the samples. The composite tested at RT for 60 h showed that the flexural strength exhibited a notable increase, while the modulus and brittle fracture behavior revealed only minor changes. In the material tested at 1000 °C for 60 h, both the modulus and strength of the sample decreased just a little, while remaining brittle. However, Testing the sample at 1300 °C for 7 h resulted in improved strength and toughness values, and the E-modulus decreased clearly.

The influence of temperature on the fatigue damage is based on its impact on internal stress, as reported in reference [13]. In the case of SiC-coated C/SiC composites, the axial and radial coefficients of thermal expansion (CTE) of the C fiber are −0.1 to −0.26 × 10^−6^ K^−1^ and 7 × 10^−6^ K^−1^, respectively, while the CTE of the SiC matrix is 4.8 × 10^−6^ K^−1^. The mismatch of the axial and radial CTEs between the C fibers and the SiC matrix generate internal stresses. Upon cooling the composites from the preparation temperature (1000 °C) to RT, microcracks were found in the matrix under axial tensile internal stress, and the interlayer between the fiber and matrix exhibited a debonding trend under radial tensile internal stress. Upon a subsequent increase in temperature, the axial tensile stress on the matrix decreased, and the microcracks in the matrix healed at a slow rate. It has been reported that cracks in 3D braided C/SiC heal at 900 °C [16]. At temperatures above 900 °C, the C fibers experience axial shrinkage, while the SiC matrix undergoes axial elongation. Consequently, the C fibers are subjected to axial tensile stress, while the SiC matrix is under axial compressive stress. Conversely, as the radial expansion of the matrix is less than that of the fiber, the internal stress on the interlayer between fiber and matrix transitions from tensile stress to compressive stress.

In combining Figure 3b and Figure 3d, it is demonstrated that the degradation mechanism of the TI C/SiC sample is analogous to that of the common C/SiC composite, as reported in reference [11]. This finding indicates that the damage of TI C/SiC also originates from the crossing points of the fiber bundles. The matrix at these points is damaged by shear stress under fatigue stress, resulting in a higher density of microcracks in the matrix close to the crossing points than that in the matrix outside the fiber bundles [16]. Consequently, the angles between the fiber bundles decrease and the fiber bundles straighten along the loading direction. This behavior results in a quick increase in the length of the sample during the first 0.5~3 h. The growth of microcracks in other regions of the matrix cause a gradual increase in the load-bearing area, which lead to a reduction in length. The residual axial compressive internal stress in the fiber impedes the propagation of cracks toward the fibers, while the residual radial tensile stress in the interlayer induced delamination of the interlayer. Consequently, the microcracks deflect and spread in the interlayer along the fiber direction, reducing the interlayer bonding strength. This phenomenon can be exploited to enhance the flexural strength of the sample. Nevertheless, the weak changes in the E-module indicates that only a limited number of microcracks are spread in the interlayer during the fatigue process.

As illustrated in Figure 3c, when the test temperature is in close proximity to the preparation temperature of 1000 °C, the matrix, fiber and interlayer are almost free of residual internal stress. Caused by the external stress, both the growth of microcracks in the matrix and the damage to the crossing sites of the fiber bundles remain. And the total length change at 1000 °C still exhibits a prolonged adjustment period followed by a fast elongation. In the absence of residual internal stress, the reduction in the load-bearing area followed by the stress redistribution is accelerated by the microcracks spreading across the interlayer rather than spreading along the fibers. The reduction in the load-bearing area causes an increase in the sample length, while the stress redistribution leads to the decrease in the sample length. This behavior occurs in all fiber bundles continuously, resulting in a frequent adjustment of the sample length during fatigue. The fiber damage leads to a decrease in the E-modulus and strength of the sample, as shown in Figure 3d.

At 1300 °C, all the existing microcracks are closed because of the thermal expansion of the SiC matrix before applying the fatigue stress. Moreover, the matrix is subjected to residual compressive internal stress, while the fiber is subjected to residual tensile internal stress, and the interlayer is exposed to both axial tensile internal stress and radial compressive internal stress. Consequently, during the fatigue process, new microcracks mainly occur inside the interlayer, and then spread inside the interlayer along the fibers or deflect to damage the fibers. This feature, on the one hand, causes the quick short-term length adjustment of the sample. On the other hand, it gives more space for the thermal expansion of the matrix, causing a longer total length, as shown in Figure 3a. The interlayer cracking, accompanied with the serious sliding friction of the interlayer, results in a reduction in the interlayer bonding strength, resulting in an improvement in the toughness and, thus, in a quasi-plastic fracture behavior of the sample, as illustrated in Figure 3d.

The initial shortening during the first 0.5 h is attributed to an apparent hardening, a phenomenon previously observed in other composites [17]. During loading, the elongation of the sample can be estimated as follows:

(1) $$\Delta l=\frac{{PL}_{1}}{E_{1}A_{1}}+\frac{{PL}_{2}}{E_{2}A_{2}}$$

where ∆*l* is the elongation (mm), *P* is the external load (N), *L* is the length (mm), *E* is the elastic modulus (GPa), *A* is the cross-sectional area, subscript 1 is the gauge section, and subscript 2 is the non-gauge section. During the fatigue test, the load is controlled; therefore, P is constant. For a particular sample, L is also constant. Once the temperature is settled, the length change in the sample and the whole test system caused by temperature is constant. When the temperature is kept steady during the test, the length change in the sample and the whole test system originated by the temperature can be ignored. Therefore, the influence of temperature on ∆*l* in Equation (1) is discounted. The phenomenon of the apparent hardening is related to the increasing elastic modulus of the composites, which is based on the hypothesis that an external loading is shared by all fibers. In this context, the decreased length change is explained by an apparent hardening. However, it should be noted that the characteristic parameter E will not change under a short-term stress. Even if it does change, it will decrease and lead to an elongation of the sample. Consequently, the sole explanation for the shortened phenomenon of the sample is the increase in the load-bearing area A. Due to the characteristics of the CVI process, the composites exhibit a density gradient of outside being higher and inside being lower [16]. At the beginning of the loading, only a portion of the cross-sectional fibers and matrix shared the load, resulting in a significant elongation of the specimen. Following a period of load transfer and stress redistribution, the cross-sectional area that shared the external load increases. The increase in the load-bearing area shortens the sample due to the apparent hardening. Then, negative displacements occur because the starting displacement was set to zero. Since A_2_ is 2.67 times larger than A_1_, even for the condition of 90 ± 30MPa, the maximum stress bore by A_2_ amounts to only 45 MPa, which is less than the crack opening stress of 60 MPa. Moreover, L_2_ is set at low temperatures during the test; therefore, oxidation effects are negligible. Consequently, the elongation of L_2_ can be disregarded, and Equation (1) has to be revised as follows:

(2) $$\Delta l=\frac{{PL}_{1}}{E_{1}A_{1}\left(t\right)}$$

where *A_1_*(*t*) is the load-bearing area in the gauge part changing with time.

### 3.2. Effect of Oxidation on the Fatigue Degradation

**Figure 4:**
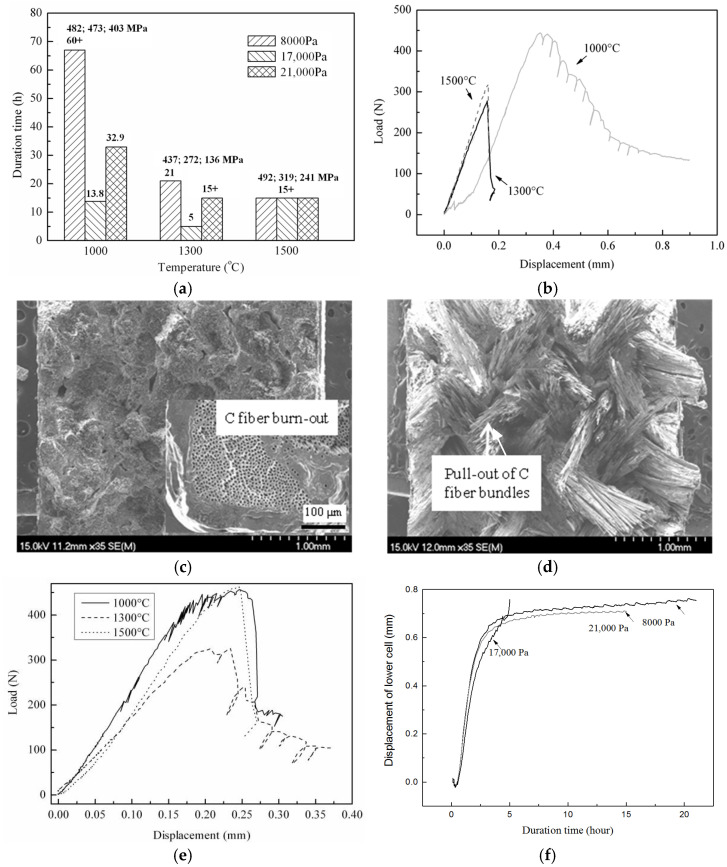
The effect of oxidation on the fatigue degradation of TI C/SiC under 60 ± 20 MPa in different dry oxygen atmospheres at different temperatures: (**a**) oxidation time and residual flexural strength, (**b**) flexural load–displacement curves of unbroken gauge after exposing the sample in an oxygen partial pressure of 17,000 Pa, (**c**) SEM image of the total fracture cross-section and near-surface oxidation cross-section of unbroken gauge after testing in an oxygen partial pressure of 17,000 Pa at 1300 °C for 5 h, (**d**) SEM image of the total fracture cross-section of unbroken gauge after testing in an oxygen partial pressure of 17,000 Pa at 1000 °C for 21 h, (**e**) flexural load–displacement curves of unbroken gauge after loading the composite in an oxygen partial pressure of 8000 Pa, and (**f**) length change in the loaded sample in different dry oxygen atmospheres at 1300 °C.

The effect of oxidation on the fatigue degradation of TI C/SiC under a 60 ± 20 MPa tensile load in different dry oxygen atmospheres at different temperatures is shown in Figure 4. The mark “+” in Figure 4a means that the sample did not fail even after more than 67 h or 15 h under the given load. According to Figure 4a, it should be noted that the residual flexural strength at each temperature decreases with the increasing oxygen partial pressure and is not related to the oxidation time. This behavior is derived from the fact that the strength degradation or the C phase oxidation is controlled by the inward diffusion of oxygen, which just depends on the concentration difference rather than on the time of exposure to oxygen. According to Figure 4b,c, the sample exposed to an oxygen partial pressure of 17,000 Pa at 1300 °C for 5 h reveals a typical brittle fracture curve during the three-point bending test and a characteristic brittle fracture cross-section because of serious C phase oxidation. However, when the temperature decreases to 1000 °C, the reaction speed between PyC phase and oxygen reduces, and the oxygen can diffuse further into the center of the sample. In turn, the PyC interlayers in the interior of the sample become oxidized, and the bonding strength is reduced. This phenomenon finally leads to a clear ductile fracture behavior (see Figure 4b) evidenced by a typical ductile fracture cross-section (see Figure 4d) and results in an enhanced fracture strength. During the three-point bending test, both fiber bundle pull-out and filament pull-out, are clearly visible, as shown in Figure 4d. When the oxygen partial pressure decreases from 17,000 Pa to 8000 Pa at 1300 °C, the sample also becomes ductile (see Figure 4e). In the case that the oxygen partial pressure and temperature decrease simultaneously, the reaction speed becomes too low to severely oxidize all the PyC interlayers. Therefore, the sample exposed to oxygen at 1000 °C with a partial pressure of 8000 Pa is just strengthened but not toughened. As displayed in Figure 4a, the lifetimes or duration times indicate the oxidation behaviors of the weakest cross-section of the samples during the tests. The weakest cross-section with the widest microcracks exhibits the most serious oxidation degradation of the C phase including the PyC interlayer and C fiber. The degree of oxidation of the PyC phase also depends on the competition between the reaction speed of C with oxygen and the diffusion speed of oxygen through the microcracks. According to Figure 4f, the oxidation of the PyC phase and the slide friction of the interlayer together make the sample length adjust short-termly and periodically. These phenomena are attributed to the alternate appearance of (i) the reduction in the load-bearing area and (ii) the stress redistribution. The former causes an increase in the sample length, while the latter decreases the sample length.

According to Figure 4b,e, all the samples tested at 1500 °C show a brittle fracture behavior independent from strength increases and decreases. This finding is attributed to the closure of the microcracks caused by the thermal expansion phenomena with the result of a slower diffusion speed of oxygen.

**Figure 5:**
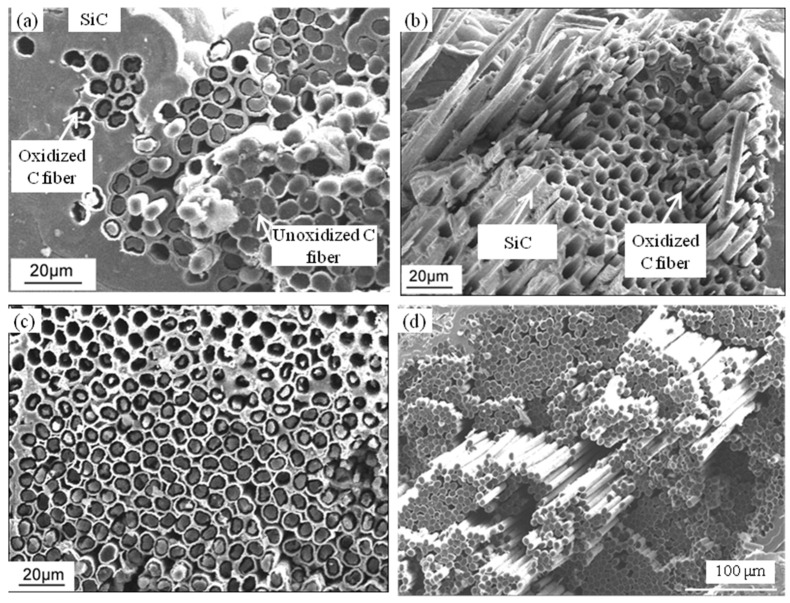
Near-surface SEM fractogram of TI C/SiC after annealing at 1500 °C for 15 h under 60 ± 20 MPa load in oxygen partial pressures of (**a**) 8000 Pa, (**b**) 17,000 Pa and (**c**) 21,000 Pa; (**d**) SEM image of the fracture surface of a sample exposed to an oxygen partial pressure of 8000 Pa.

Figure 5 shows a near-fracture-surface SEM image of TI C/SiC annealed at 1500 °C for 15 h under a 60 ± 20 MPa load in oxygen partial pressures of 8000 Pa, 17,000 Pa and 21,000 Pa. According to Figure 5a–c, the oxidation depth increases with increasing oxygen partial pressure, which is consistent with the change in the residual flexural strength. The faster reaction speed of the PyC phase with oxygen and the slower diffusion speed of oxygen together causes the oxidation to mostly occur near the surface, as shown in Figure 5a,d. The strength enhancement at a lower oxygen partial pressure is attributed to the weakening of the interface due to the stronger slide friction of the interlayer, causing fiber bundle pull-out as evaluated using the SEM image of the fracture surface seen in Figure 5d.

### 3.3. Effect of Stress Value on the Fatigue Oxidation Degradation

It has been demonstrated that tension–tension fatigue can (i) promote the extension of internal microcracks, (ii) reduce the interlayer bonding strength and (iii) enhance the strength and plasticity of TI C/SiC. Furthermore, the oxidation of the PyC interlayer also reduces the interlayer bonding strength and improves the strength and plasticity of TI C/SiC. Additionally, tension–tension stress increases the width of the microcracks, exposing more of the PyC phase to the oxidizing environment and accelerating its oxidation. However, at temperatures above 1000 °C, the matrix of the C/SiC composite is under compressive stress, resulting in the closure of the microcracks. In order for microcracks to reopen, the additional tensile stress must first overcome the internal stress. Once the microcracks reopen, the width of the microcracks will decrease due to the expansion of the SiC matrix. At a specific temperature, the internal stress and expansion of the matrix are fixed, and the width of the microcracks is directly related to the applied stress. The width of the microcracks as well as the additional tensile stress are crucial factors influencing the oxidation mechanism of the PyC phase. Therefore, a detailed investigation of the minimum amount of stress to reopen the microcracks is required.

**Figure 6:**
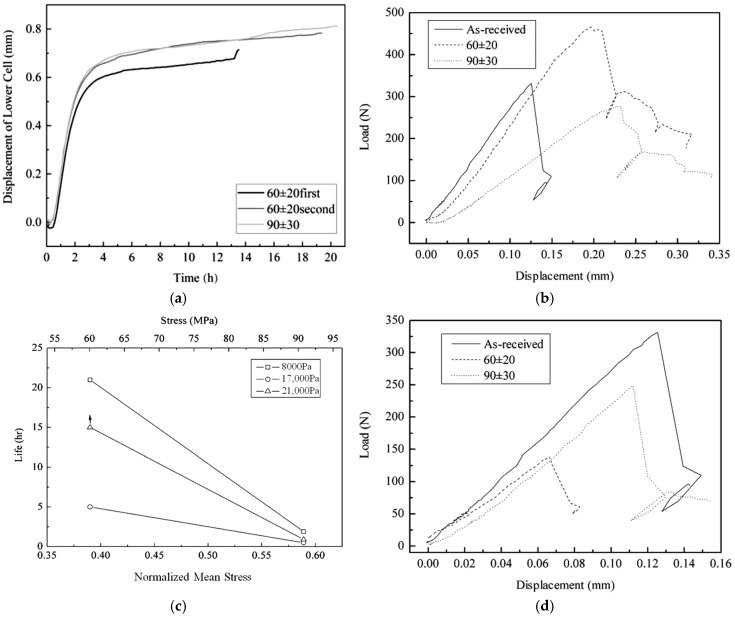
The effect of stress value on the fatigue oxidation behavior of TI C/SiC: (**a**) length change during fatigue in an oxygen partial pressure of 21 kPa at 1000 °C, (**b**) flexural load–displacement curves of unbroken gauge after annealing in an oxygen partial pressure of 21 kPa at 1000 °C, (**c**) the effect of the stress value on the oxidation lifetime in different oxygen atmospheres at 1300 °C and (**d**) flexural load–displacement curves of unbroken gauge after testing the sample in an oxygen partial pressure of 21 kPa at 1300 °C.

The influence of the stress value on the oxidation of TI C/SiC during fatigue in different oxygen atmospheres at 1000 °C and 1300 °C is illustrated in Figure 6. The total 60 h test at 1000 °C under 60 ± 20 MPa was completed in two steps; the respective curves are denoted as 60 ± 20 first and 60 ± 20 s, respectively, as illustrated in Figure 6a. In the steady state stage of the length change, the change trend in length at 1000 °C under 60 ± 20 MPa is identical to that under 90 ± 30 MPa. The two samples reveal a quick elongation at a similar oxidation time. Furthermore, for the sample loaded under 60 ± 20 MPa, the initiate length of the steady state stage during the second test run was nearly identical to that of the final pause length of the first run. The repeated length changes indicate the hereditary and periodicity of the fatigue oxidation degradation of TI C/SiC, meaning that the periodic structure of TI C/SiC also results in the transformation of the bearing area after the initial bearing area has been irreversibly damaged by fatigue oxidation.

It is notable that besides one quick elongation, the length changes in the steady state stage of all samples in an oxygen partial pressure of 21,000 Pa at 1000 °C are consistent and smooth and exhibit a similar trend to that obtained at RT rather than to that obtained at 1000 °C in Ar. As discussed in Section 3.1 and Section 3.2, the length change in the steady state stage depends on the damage behavior of the C fiber and the PyC interlayer. At 1000 °C, the residual internal stresses on the fibers, the interlayer and the matrix are almost zero. The length change is caused by two reasons: the oxidation of the PyC along the fiber and the oxidation of the PyC toward the fiber. The former reduces the interlayer bonding strength and increases the strength and plasticity of the material. The latter reduces the load-bearing area, decreases the strength and enhances the elongation of the sample. Figure 6b indicates that the fatigue oxidation makes the brittle TI C/SiC become quasi-plastic, suggesting that the interlayer bonding strength has been reduced. The lower strength and module under 90 ± 30 MPa load suggest that both reasons for the length change are enhanced by increasing the stress value. At 1000 °C, on the one hand, the two reasons causing the length change coincide, resulting in a balance between elongation and shrinkage. On the other hand, the oxidation of the PyC along the fiber hinders microcracks to spread into the fiber, just like the compressive internal stress on the fiber at RT. Therefore, the length change in TI C/SiC in an oxygen partial pressure of 21 kPa at 1000 °C is smooth like that at RT.

As illustrated in Figure 6c, the life of TI C/SiC at 1300 °C exhibits a precipitous decline with the increasing stress. Because the lifetimes in different oxygen partial pressures under 90 ± 30 MPa are almost the same, the effect of oxygen partial pressure on the fatigue lifetime is much smaller under 90 ± 30 MPa than that under 60 ± 20 MPa. This behavior indicates that the degradation of the sample is controlled by the reaction speed of PyC toward the C fiber, because all of the C phase will be surrounded by the oxidizing gas when the microcracks are wide enough. The conclusion is confirmed by the strength decrease and the brittle fracture behavior, as shown in Figure 6d. The residual flexural strength of the sample tested under 90 ± 30 MPa is higher than that under 60 ± 20 MPa, suggesting that the oxidation reaction of PyC with oxygen along the fiber is increased by the stress. The reaction between the PyC phase and oxygen enhances a lot when the temperature increases to 1300 °C, demonstrating that the faster reaction of the PyC phase magnifies the effect of stress value on the fatigue oxidation degradation.

**Figure 7:**
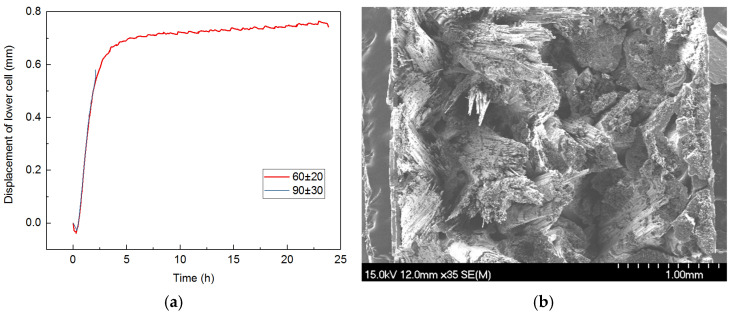

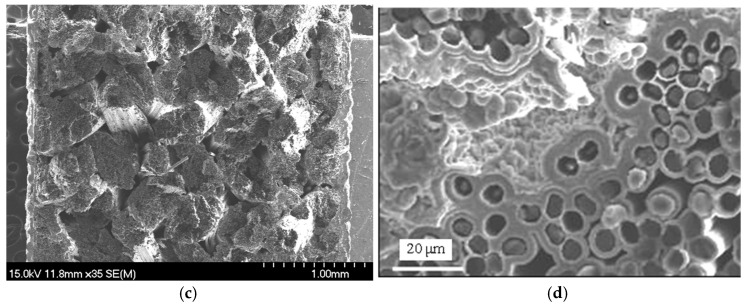
Fatigue oxidation of TI C/SiC in an oxygen partial pressure of 8000 Pa at 1300 °C: (**a**) length change during fatigue under 60 ± 20 MPa and 90 ± 30 MPa, (**b**) SEM image of the total fracture cross-section after testing under 60 ± 20 MPa, (**c**) SEM image of the total fracture cross-section after testing under 90 ± 30 MPa and (**d**) SEM image of the inner morphology after oxidation under 90 ± 30 MPa.

The fatigue oxidation of TI C/SiC in an oxygen partial pressure of 8000 Pa at 1300 °C is shown in Figure 7. According to Figure 7a, the short cycle adjustments of the length under 60 ± 20 MPa in an oxygen partial pressure of 8000 Pa demonstrate that the oxidation of the C phase is reduced at the lower oxygen partial pressure and predominantly occurs along the fibers. The oxidation of PyC along the fiber, together with the slide friction of the interlayer, makes the interlayer become debonded and causes both the fiber bundle pull-out and the filament pull-out, as seen in Figure 7b.

The larger stress value clearly accelerated the oxidation of the PyC phase, which originated the fracture of the sample during the first stage at a load of 90 ± 30 MPa, as shown in Figure 7a. However, there is still some fiber bundle pull-out identified in the total fracture cross-section shown in Figure 7c. From the SEM image of the fracture morphology, fibers near the surface of each fiber bundle are oxidized like that displayed in Figure 7d, independent of whether the fiber bundle is located in the center or near the surface of the sample. This finding indicates that the velocity of the oxygen diffusion is faster than the oxidation of the PyC phase in the oxygen partial pressure of 8000 Pa at 1300 °C. Therefore, it is concluded that the fatigue oxidation degradation is controlled by the reaction mechanism of carbon with oxygen in this case.

## 4. Conclusions

A 3D braided C/SiC with a thin pyrolytic carbon (PyC) interlayer (TI C/SiC) has similar length change behavior to that with a standard interlayer of 200~500 nm during fatigue. The length changes in the TI C/SiC composite also exhibit inheritance and periodicity, including long and short periodic adjustment. The long periodic adjustment is attributed to the angle change in the cross-sites of the fiber bundles, while the short periodic adjustment is due to the damage of the PyC interlayer and carbon fiber.

The positive damage of the thin interlayer from friction and oxidation, which develops along the fibers, increases the load-bearing area, causing the sample to shrink. Meanwhile, it increases the strength and decreases the modulus, making the brittle sample quasi-ductile. Conversely, the negative damage of the C phase (including the PyC interlayer and fiber) evolves toward the fibers, decreasing the load-bearing area, causing the sample to elongate and decreases the strength. The length change behavior of the TI C/SiC composite is contingent upon the interplay between the positive damage of the thin PyC interlayer and the negative damage of the C phase including C fibers and the PyC interlayer.

The most serious damage of the composite TI C/SiC under a load of 60 ± 20 MPa occurs in an oxygen partial pressure of 17,000 Pa at 1300 °C. With reduced oxygen partial pressure and (or) temperature, the positive damage of the thin interlayer from friction and oxidation becomes pronounced, while with increasing stress, the negative damage of the C phase toward the fibers takes over.

## Data Availability

The data presented in this study are available on request from the corresponding author due to [legal reason].
